# Comparison between Stromal Vascular Fraction and Adipose Mesenchymal Stem Cells in Remodeling Hypertrophic Scars

**DOI:** 10.1371/journal.pone.0156161

**Published:** 2016-05-26

**Authors:** Sophie Domergue, Claire Bony, Marie Maumus, Karine Toupet, Eric Frouin, Valérie Rigau, Marie-Catherine Vozenin, Guy Magalon, Christian Jorgensen, Danièle Noël

**Affiliations:** 1 Inserm, U1183, Hôpital Saint-Eloi, Montpellier, F-34295, France; 2 Montpellier University, UFR de Médecine, Montpellier, F-34967, France; 3 Chirurgie Plastique Reconstructrice et Esthétique, Hôpital Gui de Chauliac, Montpellier, F-34295, France; 4 Biopathologie, Hôpital Gui de Chauliac, Montpellier, F-34295, France; 5 Radiation Oncology Laboratory, CHUV, Lausanne, Switzerland; 6 Chirurgie Plastique, Hôpital La Conception, Marseille, F-13005, France; 7 Service d'Immuno-Rhumatologie Thérapeutique, Hôpital Lapeyronie, Montpellier, F-34295, France; Cedars-Sinai Medical Center; UCLA School of Medicine, UNITED STATES

## Abstract

Hypertrophic scars (HTS) are characterized by excessive amount of collagen deposition and principally occur following burn injuries or surgeries. In absence of effective treatments, the use of mesenchymal stem/stromal cells, which have been shown to attenuate fibrosis in various applications, seems of interest. The objectives of the present study were therefore to evaluate the effect of human adipose tissue-derived mesenchymal stem cells (hASC) on a pre-existing HTS in a humanized skin graft model in Nude mice and to compare the efficacy of hASCs versus stromal vascular fraction (SVF). We found that injection of SVF or hASCs resulted in an attenuation of HTS as noticed after clinical evaluation of skin thickness, which was associated with lower total collagen contents in the skins of treated mice and a reduced dermis thickness after histological analysis. Although both SVF and hASCs were able to significantly reduce the clinical and histological parameters of HTS, hASCs appeared to be more efficient than SVF. The therapeutic effect of hASCs was attributed to higher expression of TGFβ3 and HGF, which are important anti-fibrotic mediators, and to higher levels of MMP-2 and MMP-2/TIMP-2 ratio, which reflect the remodelling activity responsible for fibrosis resorption. These results demonstrated the therapeutic potential of hASCs for clinical applications of hypertrophic scarring.

## Introduction

Skin wound healing is a biological process that restores the epidermal and dermal layers of the skin after injuries through successive phases of inflammation, proliferation and remodeling. Abnormal healing occurs when this process is impaired resulting in chronic wounds or when it loses control ending with fibrosis or hypertrophy. Hypertrophic scars (HTS) are consequences of excessive extracellular matrix (ECM) deposition by fibroblasts and myofibroblasts and infiltration of immune cells releasing inflammatory mediators [1]. This excess of ECM occurs when the inflammatory phase is prolonged and myofibroblasts generate the scarring tissue filling the defect. Besides its undesirable visual appearance, the scar tissue presents poor mechanical strength relative to the surrounding tissue. HTS are the most common complications that may develop following burn injury and surgical incisions, and represent an important public health problem primarily in children and young people [2]. HTS is characteristic of human skin and does not develop in other animals, although mechanical load may initiate HTS in mice [3]. While there are several animal models of skin fibrosis (for review, see [4]), a small number of studies reports the use of models of HTS based on human skin [5]. One of these studies reports that transplantation of full-thickness human skin grafts leads to HTS three months after transplantation in an immunocompromised mouse [6]. Indeed, the availability of such pre-clinical models of HTS represents useful tools to address the effects of treatments.

Current treatments to promote wound healing without scar formation primarily consist in standard procedures for wound management to attenuate excessive scarring. Many of these treatments have proven some efficacy and emerging therapies are evaluating the use of recombinant TGFβ3 or anti-TGFβ1 antibodies [2]. However, an alternative to these pharmaceutical treatments could be the application of multipotent mesenchymal stem or stromal cells (MSCs). MSCs are native constituents of the wound bed [7] but are mainly isolated from bone marrow (BM), umbilical cord or adipose tissue. They are characterized by their capacity to adhere to plastic, their phenotype and trilineage differentiation potential [8]. MSCs exert numerous functions through their migratory, anti-inflammatory and trophic properties that may be of interest for restoring skin tissue function and enhance healing. By secreting anti-inflammatory mediators, MSCs influence the inflammatory phase of healing; they are also involved in bacterial clearance and the stimulation of proliferation and migration of predominant skin cell types [9]. Most of the pre-clinical and clinical studies have evaluated the interest of MSC in the treatment of acute or chronic non healing wounds [10]. However to our knowledge, there is only one report on the interest of using bone marrow-derived human MSCs to prevent HTS in the rabbit model of ear scar [11].

We therefore set up the conditions for HTS formation after the implantation of full thickness human skin grafts onto the back of immunocompromised Nude mice as a relevant model for evaluating the therapeutic potential of human adipose tissue-derived MSCs (hASCs) on HTS. We specifically aimed at evaluating the efficacy of ASC injection on the attenuation of a pre-existing HTS and comparing the local application of hASCs with that of stromal vascular fraction (SVF).

## Methods

### Isolation and characterization of adipose tissue-derived mesenchymal stem cells

Human fat tissue samples were obtained through abdominal dermolipectomy from patients undergoing plastic surgery after written informed consent signed by the patients. The study was specifically approved by the French Ministry of Research and Innovation and the Personal data Protection ethics Committee (CPP) of Languedoc-Roussillon (approval AC-2010-1200). In accordance with French law on the protection of personal data, the medical records were anonymized prior to use. Subcutaneous abdominal fat was digested with 250 U/mL of collagenase type II at 37°C for 1h. After centrifugation (300 g, 10 min), the stromal vascular fraction (SVF) was collected, filtered successively through a 100 μm, 70 μm and 40 μm porous filter (Cell Strainer, BD Biosciences, le Pont-de-Claix, France). Quantification of viable cells was performed using a hemocytometer after Trypan blue staining and frozen in liquid nitrogen. Freshly thawed SVF was immunophenotyped and immediately used for animal experimentation.

For ASC isolation, SVF prepared freshly as described above was plated at 4000 cells/cm^2^ in α-MEM medium supplemented with 100 U/mL penicillin/streptomycin, 2 mmol/mL glutamine and 10% fetal calf serum. After one week in culture (end of passage 0), adherent fibroblastic hASCs were characterized by their immunophenotype (positive for CD13, CD73, CD90, CD105 and negative for CD14, CD31, CD34, CD45) (S1 Fig), their trilineage potential of differentiation as published [12] and frozen. For the experiments, hASCs were thawed, expanded in culture for 1 week and used before 14 population doublings (end of passage 1).

### Flow cytometry analysis

SVF (100 μL) or hASCs (1×10^5^) in PBS containing 0.2% bovine serum albumin (BSA) were incubated with 1 μL of antibodies for 20 min at 4°C in the dark. Antibodies tested were: CD13, CD14, CD31, CD34, CD45, CD73, CD90 or CD105 antibodies or the respective isotype controls (BD-Biosciences). The labeled cells were then analyzed by multiparameter flow cytometry using a FACSCanto cytometer and the Diva software (BD-Bioscience).

### Skin samples

Full thickness skin (1.5 mm in thickness), which corresponded to the epidermis and dermis layers, without the underlying hypodermis, was obtained from healthy women undergoing mammary reduction after informed consent and approval by the Local Ethical Committee (registration number: LB-2012-12-01). Skins were wrapped with saline soaked gauze and placed in a sterilized instrumental box at room temperature for transport to the neighboring laboratory. Skins were grafted between 30 min to 6 hours after removal.

### Animal experimentation

Athymic Nude-*Foxn1*^*nu*^ mice aged 6–7 weeks were obtained from Harlan (Gannat, France) and housed at the animal facility in controlled conditions. Animal experimentation was conducted in agreement with the Languedoc-Roussillon Regional Ethics Committee on Animal Experimentation (approval CEEA-LR-11053). All surgery was performed under isoflurane gas anesthesia, and all efforts were made to minimize suffering and to reduce animal numbers. Animals received oral paracetamol for the first few days after graft and were monitored weekly for graft outcomes.

Skin samples from two donors were used: half of the mice were implanted with the skin of one donor and half with the skin of the other donor. Because in preliminary experiments we found out that approximately 25% of skin grafts did not succeed (essentially because only part of the graft survived), 40 mice were implanted. A circular piece of skin (2 cm in diameter) was removed on the back of mice. Human skin samples (2 cm in diameter and 1.5 mm in thickness) were grafted to fit into the prepared graft sites and fixed in position with 5.0 suture (Optim' R^**®**^ 5.0; Peters Surgical, Bobiny, France) and suture dressing (Urgotul^**®**^; Urgo Laboratory, Chenove, France). An extensive bandage was applied during 10 days after grafting to prevent graft desiccation. Clinical follow-up was performed every week for 9 weeks after grafting. Measure of skin graft thickness was performed once a week using a caliper by the measurement technique of skinfold. Briefly, the graft skin and the neighboring mouse skin were pinched between two fingers in order to overlay both skins. Measure of the graft thickness took into account both the human and mouse skins. The mouse skin thickness being low and similar between mice, most of the differences were accounted for the human grafts.

At week 7 after implantation, 30 mice were selected after visual examination of the grafts and mice were separated in 3 groups of 10 mice with homogeneous skin grafts. Grafts were treated with phosphate buffered saline (PBS), hASCs or SVF. The day of injection, the quantity of hASCs contained in a freshly thawed SVF was determined by flow cytometry. Cells positive for CD13, CD34, CD73, CD90 and negative for CD14, CD31, CD45 were considered hASCs and accounted for 45% of the cells in the SVF. SVF containing 10^6^ hASCs (100 μL) was injected subcutaneously in the scar in the 4 cardinal points in one group (n = 10). A total of 10^6^ cultured hASCs in 100 μL was injected in the second group (n = 10) and 100 μL of PBS was injected into the control group (n = 10). At week 8, 5 mice/group were euthanatized and the remaining 5 mice/group were euthanatized at week 9. Euthanasia was performed by CO_2_ inhalation. The day of sacrifice, scar thickness was measured with a caliper. Skin graft punches (6 mm in diameter) were recovered and either frozen at -80°C for RNA and collagen analysis or fixed in 3.7% formaldehyde for histology.

### Histological analysis

Skin samples were embedded in paraffin and cut in sections of 5 μm, stained with Masson’s Trichrome for analysis of collagen-rich and fibrotic areas. Histological sections were scanned using a nanozoomer digital slide scanner (Hamamatsu, Massy, France). Mean skin thickness was calculated on more than 30 points from each section using ImageJ skin tool plugin. Results are expressed in μm. Fibrosis and scar were quantified on skin sections as follows: demarcation between papillary and reticular layers was scored 1 when it could not be evaluated and 0 when clearly preserved; presence of dense collagen fibers and disorganization in collagen bundles was scored 1 or 0 when absent or normally organized; disorganization in elastic fibers including fragmented, agglutinated fibers was scored 1 or 0 for normal appearance. In addition, fibrosis was scored as follows: 0 for no fibrosis, 1 for thin fibrosis that did not concern all dermis, 2 for dense thick hyaline fibrosis. A scar score (from 0 to 7) was established based on these parameters and used to compare skin sections between groups.

Sections of paraffin-embedded samples were immunostained with antibodies for human pan-cytokeratin, cytokeratin 5/6, involucrin or α-Sma (Abcam, Paris) or stained for DAPI.

### Collagen content in skin

Collagen content assay was based on the quantitative dye-binding Sircol method (Biocolor, Interchim, Montluçon, France). Skin biopsies were suspended in 2 mL of a 0.5 M acetic acid/pepsin solution and dissociated with a dispenser tool (Ultra-Turrax IKA, VWR, Fontenay-sous-Bois). Collagen extraction was performed at 4°C overnight under stirring. The suspension was then centrifuged at 12,000 g for 10 min. Part of the supernatant (20 μL) was used for total protein quantification using the BCA assay (Sigma) and another part (20 μL) of the sample was added to 1 mL of Syrius red reagent. After 30 min agitation at room temperature, samples were centrifuged at 12,000 g for 10 min and supernatants discarded. Pellets were washed with 750 μL of ice-cold salt acid and centrifuged at 12,000 g for 10 min, before resuspension in 1 mL of 0.5 M NaOH Alkali solution. Optical density was then read at 555 nm on a microplate reader (Varioskan Flash, Thermo Scientific, Courtaboeuf, France) versus a standard range of bovine collagen type I concentrations (supplied as a sterile solution in 0.5 M acetic acid). Results were expressed in mg of collagens/mg of total proteins.

### RNA extraction and real time RT-PCR

Skin punches were mechanically dissociated using a dispenser tool (Ultra-Turrax IKA, Imlab, Lille, France). Punches were suspended in lysis buffer for dissociation and RNA extraction was performed using the RNeasy mini kit and Qiacube robotic workstation as recommended by the supplier (Qiagen, Courtaboeuf, France). Quality and quantity of RNA were checked on a NanoDrop spectrophotometer (Thermo Scientific, Villebon sur Yvette, France). One μg RNA was reverse transcribed using 100 units of the Moloney Murine Leukemia Virus Reverse Transcriptase (M-MLV RT, Invitrogen). Reverse Transcription conditions were as follow: 25°C for 10 min; 37°C for 50 min and 70°C for 15 min. Real time PCR (qPCR) was performed on 20ng RNA in 10μL that contained 5μl of DNA Master SYBR Green I kit (Roche Diagnostics, Meylan) and 0.05μM primers. Sequences of primers were designed using the web-based applications Primer3 and BLAST and reported in Table 1 (). The following PCR conditions were used: 95°C for 5min; 40 cycles at 95°C for 15s; 64°C for 10s and 72°C for 20s in a LightCycler 480 system (Roche diagnostics, Meylan, France) and analyzed with the dedicated software. Results were provided as relative expression to the housekeeping gene tyrosine 3-monooxygenase/tryptophan 5-monooxygenase activation protein, zeta (YWHAZ) using the formula 2^−ΔCt^.

**Table 1:** List of primers used in RT-qPCR experiments.

Gene abbreviation	Forward primer sequence (5’-3’)	Reverse primer sequence (5’-3’)
*aSma*	CATCGGGATGGAGTCTGCTG	AGAAGCATTTGCGGTGGACA
*Col1*	CCTGGATGCCATCAAAGTCT	CGCCATACTCGAACTGGAAT
*Col3*	CGCCCTCCTAATGGTCAAGG	AGGGCCTGAAGGACCAGCTT
*Hgf*	CCCCATCGCCATCCCCTATG	ACATCTATTAGCACATTGGTCTGCACT
*IFNγ*	ATGTCCAACGCAAAGCAATACATGAAC	ACCTCGAAACAGCATCTGACTCCT
*IL6*	TTCTCCACAAGCGCCTTCGGTC	GAATCTTCTCCTGGGGGTACTGGGG
*Mmp1*	AGGCCCAGGTATTGGAGGGGA	GCCGATGGGCTGGACAGGATT
*Mmp2*	GCCGTCGCCCATCATCAAGTT	ATAGAAGGTGTTCAGGTATTGCACTG
*Mmp3*	TTGCGCCAAAAGTGCCTGTCT	GGAGCCAGGCTTTCCCAAGCA
*Mmp9*	CAAGGGCGTCGTGGTTCCAA	GGCCCTCGAAGATGAAGGGGAAGT
*Mmp13*	TAAGGAGCATGGCGACTTCT	GTCTGGCGTTTTTGGATGTT
*Tgfb1*	ACTGCAAGTGGACATCAACGGGTTCAC	GCCATGAGAAGCAGGAAAGGCCGGT
*Tgfb3*	CTAAGCGGAATGAGCAGAGGATC	TCTCAACAGCCACTCACGCACA
*Timp2*	AGGGCCAAAGCGGTCAGTGA	AACGTCCAGCGAGACCCCAC
*Tnfa*	AGCCCATGTTGTAGCAAACCCTC	TGGTTATCTCTCAGCTCCACGCCA
*VegfA*	CTTGCTGCTCTACCTCCACC	ATGATTCTGCCCTCCTCCTT
*Ywhaz*	AGTTAAGGGCCAGACCCAGT	AGACGGAAGGTGCTGAGAAA

*aSma*: *α-*smooth muscle actin; *Col*: collagen; *Hgf*: hepatocyte growth factor; *Ifnγ*: interferon γ; *IL6*: Interleukin 6; *Mmp*: matrix metalloproteinase; *Tgfb*: transforming growth factor β; *Timp*: tissue inhibitor of metalloproteinase; *Tnfa*: Tumor necrosis factor α; *Vegf*: vascular endothelial growth factor; *Ywhaz*: Tyrosine 3-monooxygenase/tryptophan 5-monooxygenase activation protein, zeta

### Statistical analysis

Statistical analysis was performed with GraphPad Software (San Diego, CA). Comparison between groups used Shapiro-Wilk normality test. Data were compared using the Mann-Whitney’s test for nonparametric values. A p value ≤ 0.05 was considered significant.

## Results

### Full-thickness human skin graft induced hypertrophic scar formation in Nude mice

**Fig 1:**
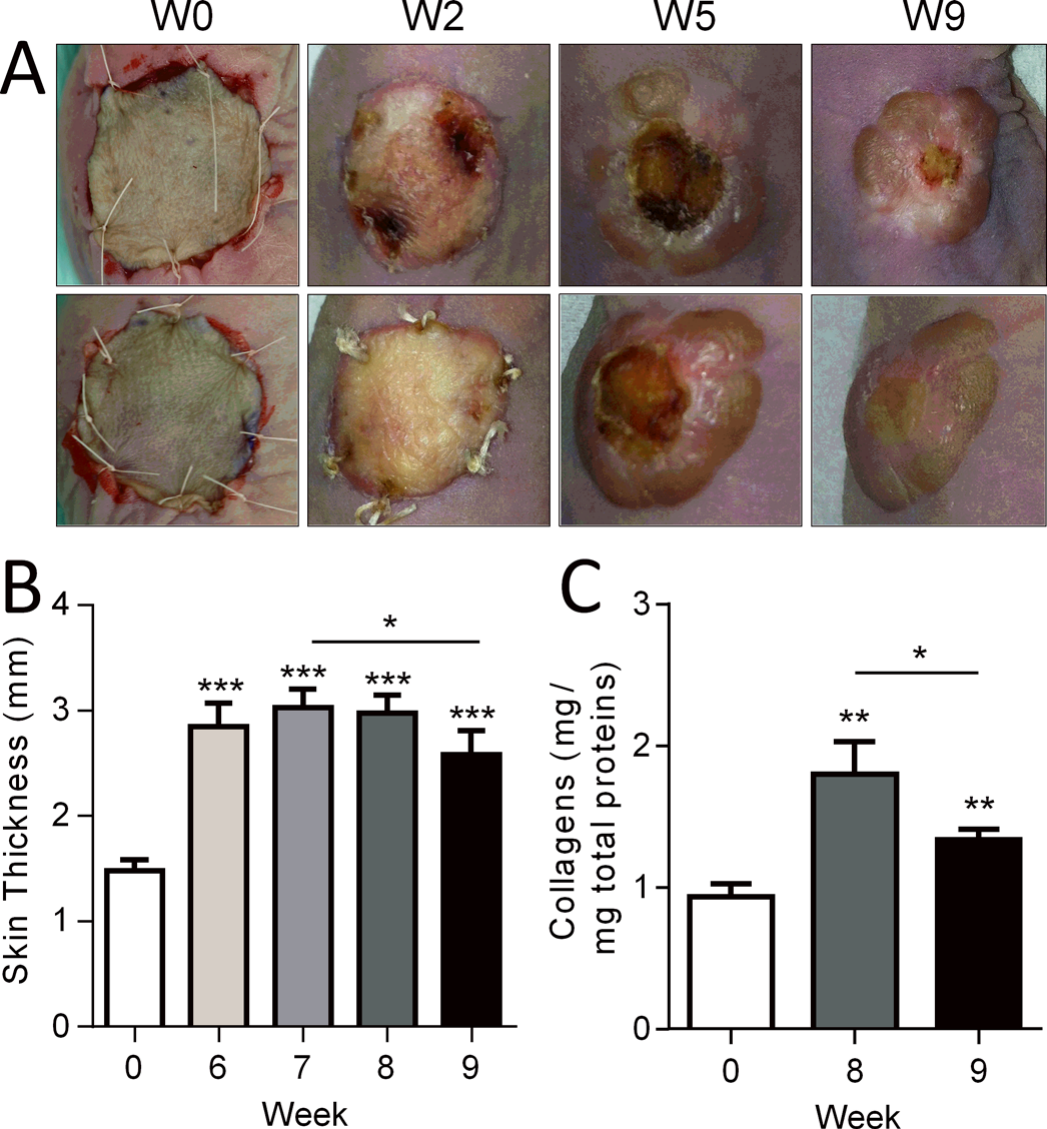
Human skin grafts in a humanized mouse model. (A) Macroscopic images of two examples of human skins (up and down panels) grafted onto the back of two immunocompromised Nude mice at different time points. (B) Measures of human skin graft thickness at different time points. (C) Quantification of collagen content in skin grafts at euthanasia. Results are expressed as the mean ± SEM (standard deviation of the mean), n = 5/group. *p<0.05, **p<0.01, ***p<0.001.

We first aimed at setting up the conditions for hypertrophic scar formation after implantation of human skin grafts onto the back of immunodeficient mice in which a piece of skin of comparable diameter has been removed. We therefore tested the implantation of human full-thickness skin grafts that were sutured to the mouse skin (Fig 1A). Two weeks after grafting, the superficial layer of the graft was not viable as indicated by white, thick and retractile overlaying tissue. Grafts then became brown and black before necrosis falling down slowly. When necrotic areas appeared in the central zone, a raised bead formed in the peripheral zone around week 5 (Fig 1A). Healing then progressed from the periphery to the central zone while a raised scar developed.

During these healing phases, we measured the skin thickness from week 6 to week 9, when the necrotic zone has disappeared. Compared to the thickness of skin at implantation (day 0), a high and significant increase of skin thickness was observed at week 6, that plateaued at week 7 and 8 (Fig 1B). Although still higher than normal skin (week 0), skin thickness was however significantly reduced at week 9 as compared to week 7, indicating a spontaneous resorption of hypertrophy. Monitoring the collagen content in skin biopsies recovered at week 8 and 9 confirmed that fibrosis with high collagen deposits occurred in the grafts with time (Fig 1C). In concordance with skin thickness decrease, a significant reduction of collagen content was observed in skins biopsied at week 9 as compared to week 8.

**Fig 2:**
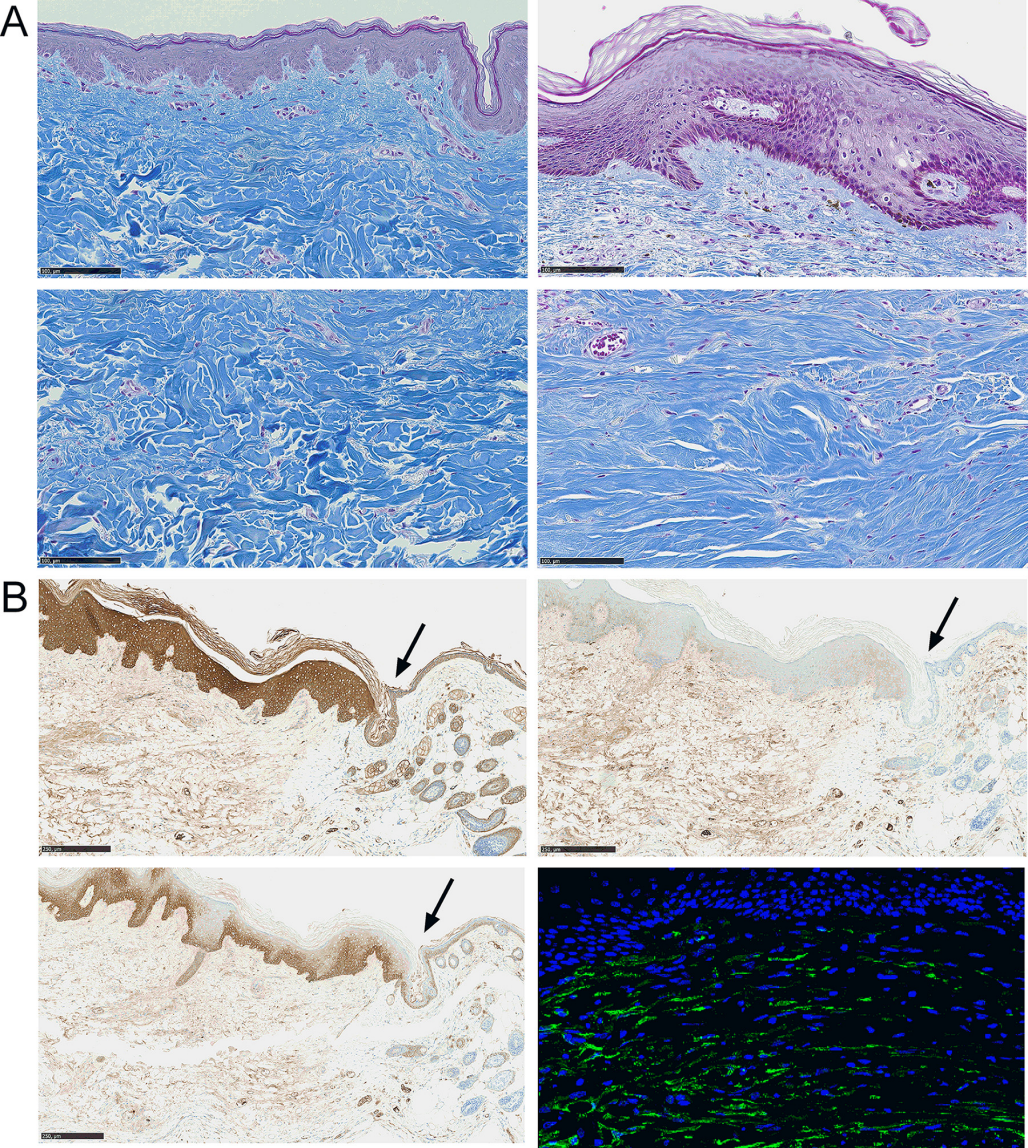
Hypertrophic scar formation in a humanized mouse model. (A) Masson’s trichrome staining of control human skin grafts (Ctrl; left) or hypertrophic scar (HTS; right) at week 8. Pictures focused on epidermis (upper panels) and dermis (lower panels) at same magnification (scale bar is 100 μm). (B) Immunohistochemical staining of HTS skins using antibodies specific for human pan-cytokeratin (upper left panel), human cytokeratin 5/6 (lower left panel), human involucrin (upper right panel) (scale bar is 250 μm). Immunofluorescence using an antibody specific for α-Sma (lower right panel) (magnification x40). Arrow indicated the demarcation between human (left) and mouse (right) tissues.

Histological analyses of skin biopsies performed at week 9 compared to normal skin indicated thickening of the epidermis and dermis layers. The most striking effect was observed in the epidermal layer, which was thicker in HTS skins than in control skins (Fig 2A, upper panels). In HTS samples, dermis was disorganized (Fig 2A, lower panels). The demarcation between the papillary and reticular layers of dermis was imprecise. In the dermis, a scar tissue with thick hyaline fibrosis was observed. Collagen fibers were dense, with disorganization in collagen bundles. Elastic fibers appeared spaced and fragmented.

Further characterization of skin grafts aimed at evaluating the presence of human tissue at the site of implantation. Using antibodies specific for human epidermis, we observed high staining for pan-keratin throughout the epidermal layer in the human skin while such high staining was not observed in the neighboring murine epidermis (Fig 2B, upper left panel; see the arrow indicating the demarcation between human (left) and murine (right) tissues). High staining for cytokeratin 5/6 was localized in the basal layer of human but not of murine epidermis (Fig 2B, lower left panel) while involucrin was expressed uniquely in human epidermis but at low levels (Fig 2B, upper right panel). Finally, fibrosis was characterized by high expression of α-Sma in the dermis of human HTS skins (Fig 2B, lower right panel) while scarce staining was observed in murine skin (data not shown).

**Fig 3:**
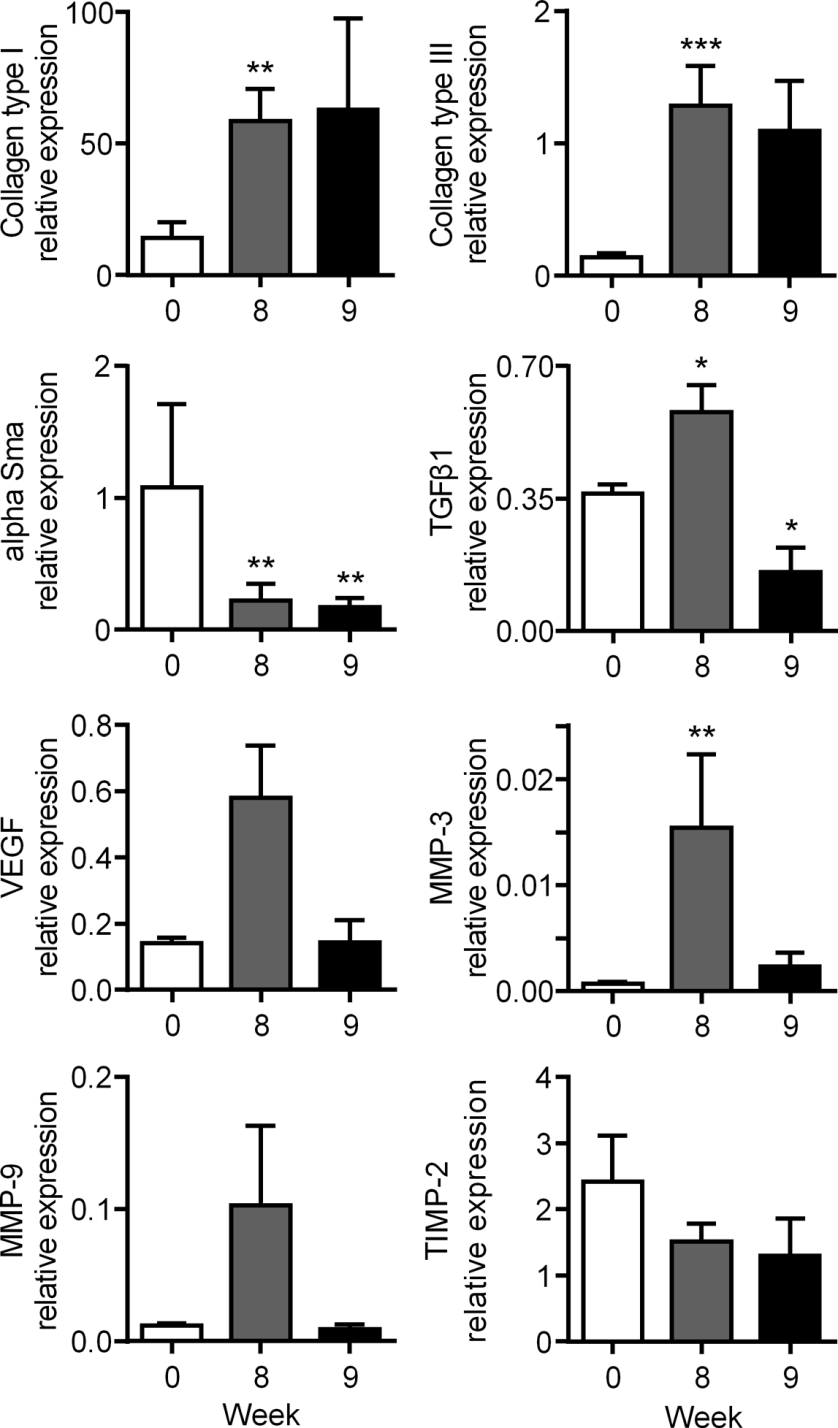
Expression of fibrotic and remodelling markers in the hypertrophic scar humanized mouse model. Measure of mRNAs of the indicated genes normalized to human *YWHAZ* expression in skin samples at weeks 8 and 9. Results are expressed as the mean ± SEM (standard deviation of the mean), n = 5/group. *p<0.05, **p<0.01.

Because skin thickness was maximal at week 8 after graft implantation and decreased at week 9, we evaluated the expression levels of markers characteristic of fibrotic scar at these two time points. The expression of transcripts for collagen type I and III were highly upregulated at weeks 8 and 9 as compared to day 0 (time of skin implantation) while α-Sma was significantly reduced at both time points (Fig 3). Expression of TGF-β1 was significantly increased by week 8 and reduced by week 9. Similar expression profiles were observed with the marker of angiogenesis VEGF and markers of remodeling MMP-3, MMP-9. Although TIMP-2 expression tended to decrease at both time points, the decrease was not significant. Altogether clinical, histological and molecular parameters indicated that human full-thickness skin grafts formed a hypertrophic scar between 6 and 8 weeks after implantation in Nude mice.

### Injections of SVF or hASCs improved hypertrophic scarring

**Fig 4:**
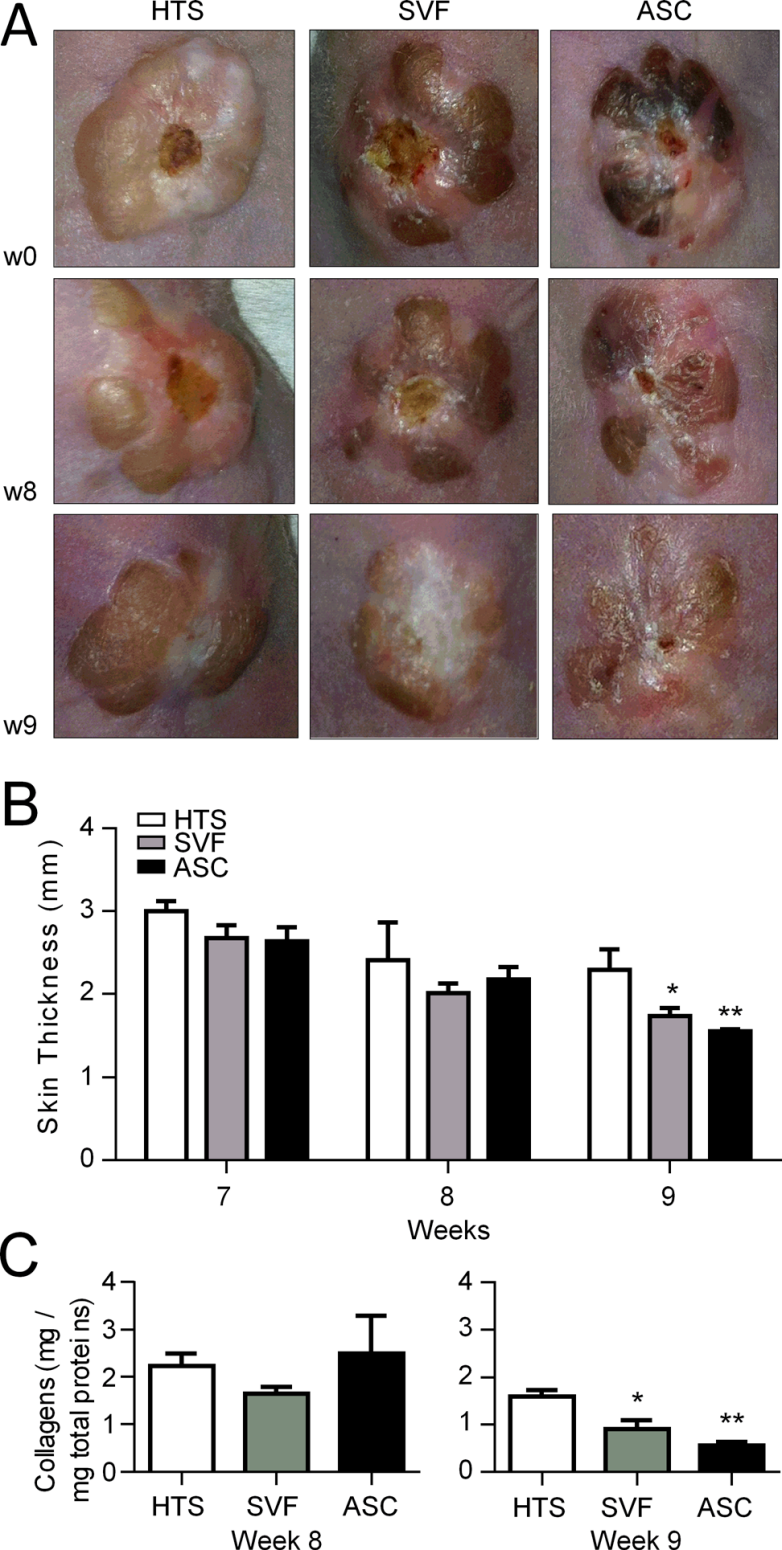
Reduction of hypertrophic scar after SVF or hASC injection in the hypertrophic scar humanized mouse model. (A) Macroscopic images of human hypertrophic scar (HTS) grafted onto the back of immunocompromized Nude mice at week 7 (w7) at the time of cell injection (upper panels). Same HTS after injection of saline (Ctrl, left), SVF (middle) or hASCs (right) at weeks 8 and 9. (B) Measures of human skin graft thickness at different time points after saline, SVF or hASC injection. (C) Quantification of collagen content in skin grafts after saline, SVF or hASC injection and measured at euthanasia (weeks 8 or 9). Results are expressed as the mean ± SEM (standard deviation of the mean), n = 5/group. *p<0.05, **p<0.01.

Using this model of hypertrophic scarring, we investigated whether hASCs could reduce hypertrophic fibrosis and compared the effect of hASCs with that of SVF. We used freshly thawed SVF and culture expanded hASCs. The day of injection, SVF preparations were immunophenotyped to determine the percentage of hASCs in the samples (S1 Fig). We injected a similar quantity of hASCs (either cultured hASCs or hASCs in the SVF suspension) in the grafts. Based on the kinetics of hypertrophic scarring, we decided to inject the cell suspensions at week 7 and to monitor different parameters 1 and 2 weeks later. At the macroscopic level, no clear difference was observed between treated and control groups at week 8 (Fig 4A). At week 9 however, scars became flatter after SVF or hASC treatment than in the control group. This was confirmed by the measure of skin thickness. One week after injection (week 8), the skin thickness tended to be lower in the treated groups as compared to the control group but with no significant difference. By contrast at week 9, a significant decrease of the skin thickness was measured in both SVF- and hASC-treated groups (Fig 4B). The reduction of skin thickness was even more significant in hASC-treated group than in SVF-treated group as compared to control. Similar results were observed with the quantification of the collagen content in the skins at euthanasia. While there was no difference in collagen content between groups at week 8, both treated groups displayed lower amounts of collagens than HTS skins at week 9 (Fig 4C). The difference was even more significant in the hASC-treated group than in the SVF-treated group.

**Fig 5:**
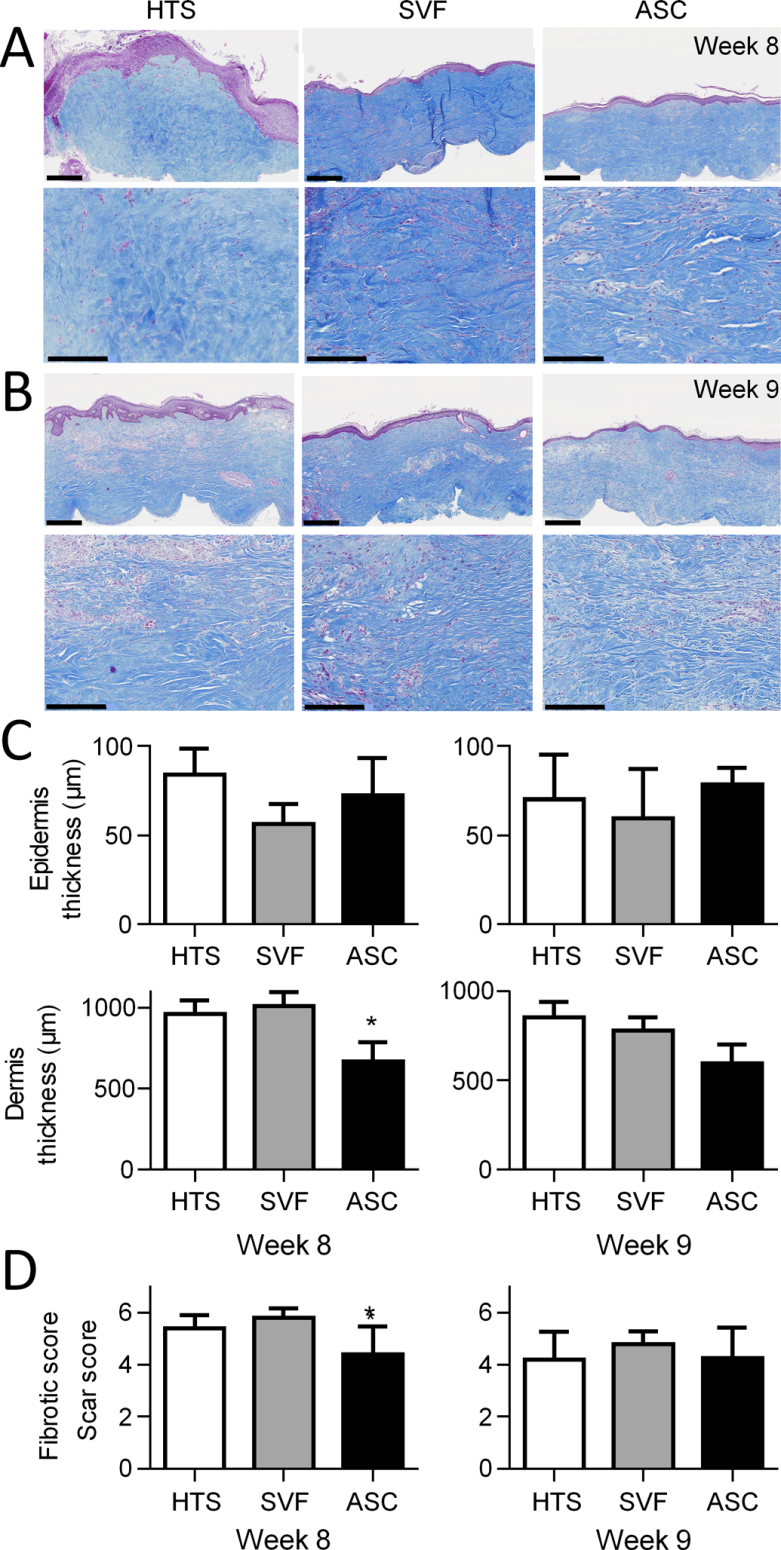
Histological evaluation of hypertrophic scar after SVF or hASC injection in the hypertrophic scar humanized mouse model. (A) Masson’s trichrome staining of control hypertrophic scars injected with saline (HTS; left), SVF (middle) or hASCs (right) at weeks 8 (scale bar is 1 mm in upper panels and 100 μm in lower panels). (B) Same as in A) at week 9. (C) Quantification of epidermis and dermis thickness on histological sections of the grafts after saline, SVF or hASC injection and measured at euthanasia (weeks 8 or 9). (D) Fibrotic score evaluated on histological sections of groups described in C). Results are expressed as the mean ± SEM (standard deviation of the mean). n = 5/group (one experiment representative of two). *p<0.05.

At the histologic level, the thickness of both epidermis and dermis were decreased in hASC- and SVF-treated groups compared to control at week 8 and 9 (Fig 5A and 5B, upper panels). This was reflected by the quantification of epidermis and dermis thickness on histological sections. A tendency to lower epidermis thickness was observed in SVF and hASC-treated groups at week 8, although differences between groups were not significant (Fig 5C). Nevertheless, dermis thickness was significantly lower in hASC-treated skin sections at weeks 8 as compared to control and tended to be still lower at week 9 (Fig 5C). Collagen fibers, which were disorganized in control groups, appeared less dense and organized in parallel bundles of fibers in the hASC-treated group (Fig 5A and 5B, lower panels). Scar score, as defined in Materials and Sections, confirmed significant reduction of fibrosis in skin sections of hASC-treated skins as compared to control mice at week 8, but not at week 9 (Fig 5D).

### SVF or hASC injections enhanced fibrosis remodeling

**Fig 6:**
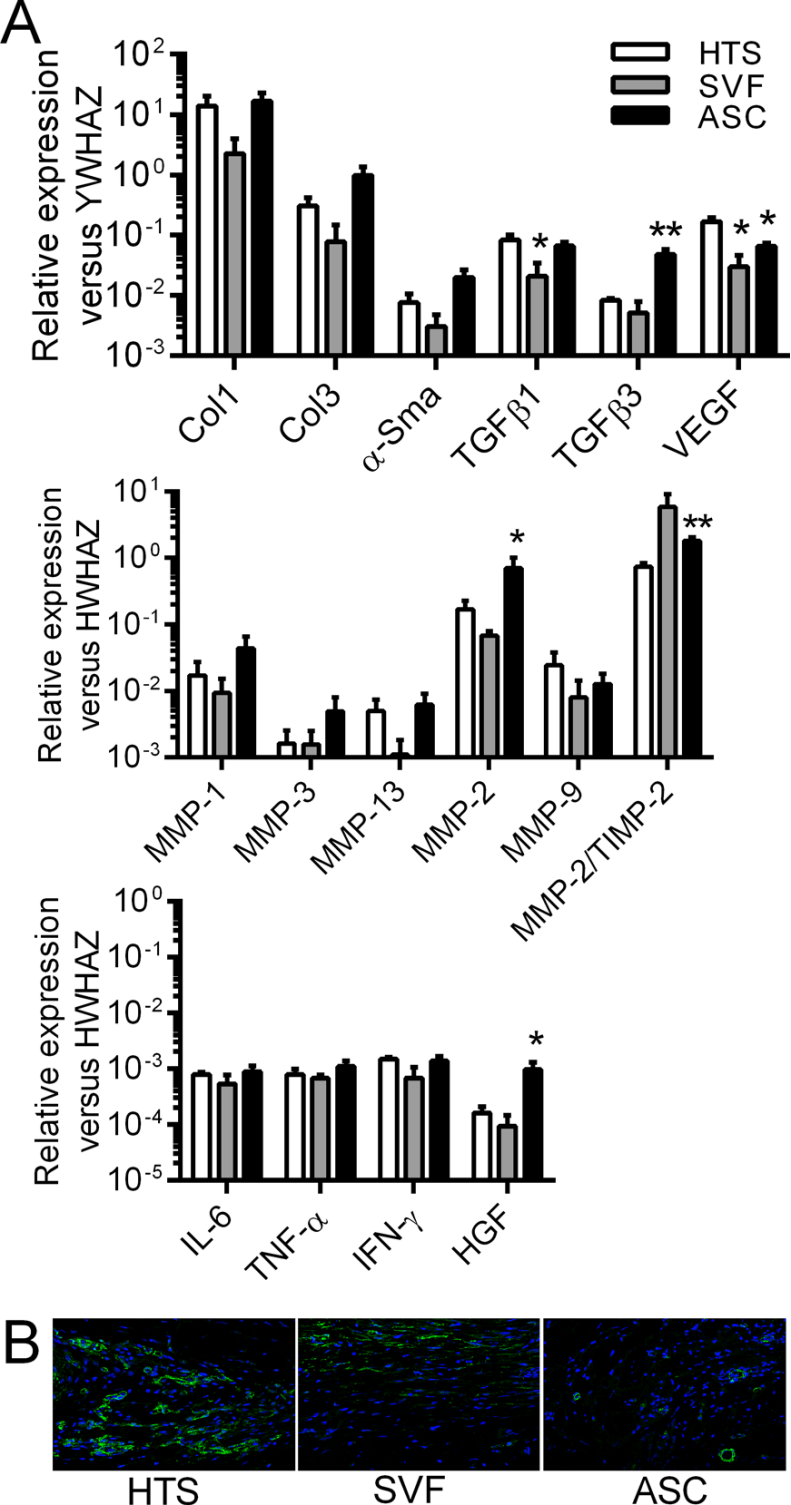
Expression of fibrotic, remodelling and inflammatory markers after SVF or hASC injection in the hypertrophic scar humanized mouse model. (A) Measure of mRNAs of the indicated genes normalized to human *YWHAZ* expression in skin samples at week 8. Results are expressed as the mean ± SEM (standard deviation of the mean). n = 5/group. *p<0.05, **p<0.01. (B) Representative pictures of α-Sma positive areas in skin grafts after saline, SVF or hASC injection at week 8 by immunofluorescence staining.

In order to determine by which mechanisms hASCs or SVF might influence the decrease in skin fibrosis, we performed transcriptomic analysis of several genes involved in fibrosis, vascularization, tissue remodeling or inflammation. At week 8, we observed no significant variations of transcript levels for the fibrotic markers collagen type I, collagen type III or α-Sma between hASC- or SVF-treated groups and control HTS groups (Fig 6A). We however detected a significant reduction of TGF-β1 expression in SVF-treated mice and of VEGF expression both in hASC- and SVF-treated groups. By contrast, the expression of TGF-β3 significantly increased in hASC-treated mice. Looking at markers involved in matrix remodeling, we did not detect any significant changes in the expression of MMP-1, -3, -9 and -13 between groups. However, we measured significantly increased expression levels of MMP-2 and the ratio MMP-2/TIMP-2 in hASC-treated group versus control group. Concerning inflammatory factors, no changes were detected between groups. Interestingly HGF, which is a well-known negative regulator of inflammatory and fibrotic markers, was highly up-regulated in hASC-treated group. At week 9, the expression levels of these different markers were similar in all the groups except for MMP-1, which was significantly increased after hASC treatment (S2 Fig). Finally, we looked at the expression of α-Sma at the protein level in the skins of control and treated groups. While several zones stained highly positive for α-Sma in the dermis of HTS skins, rare zones of α-Sma positive staining was observed in the skins of hASC- or SVF-treated skins (Fig 6B). Therefore, the efficacy of SVF and hASCs to reduce HTS at the clinical and histological level was likely related to their anti-fibrotic effect as indicated by expression of anti-fibrotic and remodeling markers.

## Discussion

In the current study, we demonstrated that the local application of human SVF or hASCs efficiently reduced skin fibrosis in a relevant model of human HTS, as shown by reduced skin thickness, lower collagen content and regulated expression of fibrotic and remodelling mediators.

We here described a humanized model of HTS following the transplantation of full-thickness human skin grafts recovered following macromastia surgery. A similar model of full thickness skin graft has been described some years ago [6]. In this last study, HTS developed at 3 months, reaching a peak in thickness by months 5–6 and then decreasing at month 8. The explanation for the shorter delay observed in our study is not known although it could be attributed to the site of skin sampling, age of the patients or skin thickness. In our conditions, HTS was maximal at weeks 6–8 and decreased thereafter. Spontaneous and partial regression of hypertrophic scars is also observed in humans over time and this characteristic distinguishes HTS from keloids [13]. In preliminary experiments, we used split thickness skin graft*s* and we observed a good healing of the grafts but no hypertrophic scar formation (data not shown) confirming that full-thickness skin grafts are required.

Previous results have described that BM-derived MSCs can prevent the formation of HTS in a rabbit model of ear scarring. In one study, the local application of human BM-MSCs reduced the thickness of granulation tissues at day 14 post-lesion and prevented scar formation [11]. The observed effects were associated with reduced inflammation mediated by secretion of TSG-6 by MSCs, which were activated by the pro-apoptotic environment. TSG-6 was shown to decrease MCP-1, MIP-1α, IL-6 and TGF-β1, inducers of inflammation and fibrosis. TSG-6 secretion was induced in pro-apoptotic caspase-3-positive MSCs. In another study, the same authors reported similar results using systemic infusion of rabbit BM-MSCs [14]. The anti-scarring function of MSCs was p53-dependent and related to the inhibition of both myofibroblast differentiation and NO production. Our results are in line with these two studies and provide further information. First, we relied on the use of a humanized model, which reproduces the main characteristics of human HTS: skin fibrosis, high collagen deposition, increased levels of fibrotic, remodelling markers as well as spontaneous but slow resorption of the scar [2]. We could not investigate the anti-inflammatory function of hASCs on cells of the adaptive immunity since we used immunodeficient mice. Of interest, this pre-clinical model allowed evaluating the therapeutic potential of human ASCs on human skin grafts, which is closer to the clinical situation. Second, we were able to show that fat-derived SVF or hASC injection can efficiently reduce a pre-existing HTS, which as far as we know, has not been previously investigated. Although the interest of SVF or hASCs has been investigated for wound healing, no data on their effect on HTS is available.

The use of SVF transplantation has been tested for soft tissue repair, for breast or face reconstruction [15]. It has also been evaluated to treat patients with radiation-induced severe burns where a systematic improvement and even remission of clinical and histological symptoms were observed in these untreatable irradiated patients [16]. More recently, autologous SVF injection in 12 patients with systemic sclerosis resulted in a significant improvement in hand disability and pain, Raynaud's phenomenon, finger oedema and quality of life [17]. The hASCs in the SVF were proposed to be the major mediators of the therapeutic effect and culture expanded hASCs are being evaluated in clinical trials [18, 19]. The interest of hASCs has been demonstrated ex vivo and in vivo in murine models of wound healing when injected into the wound or applied within a scaffold [20–22]. hASC application reduced the wound size and accelerated re-epithelialization at the wound edge. Additionally, hASC-enriched fat grafts implantation in patients for reconstructive surgery improved the survival of grafts as compared to control fat grafts [23]. On the 13 patients enrolled, those receiving hASC-enriched fat had significantly higher skin residual volumes as compared to control grafts, with no serious adverse effects. Here we provide evidence that both SVF and hASCs accelerated healing of HTS; the therapeutic effect being however more significant with hASCs than SVF. Such comparative studies on the therapeutic potential of SVF versus hASC are scarce and to our knowledge, not performed for skin healing. In a recent study, intraperitoneal administration of SVF was compared with hASC or BM-derived MSC in a murine model of autoimmune encephalomyelitis [24]. All cell preparations significantly ameliorated the clinical signs of the disease and the SVF cells were as effective as culture expanded cells in their ability to reduce disease progression. In this last study however, the quantity of hASCs and of total SVF was identical. Here, we injected the same quantity of hASCs (10^6^ cultured hASCs or SVF cells containing 10^6^ hASCs) and observed a better effect with isolated hASCs. This suggested that culture expanded hASCs have a high capacity to reduce the clinical symptoms of HTS and that other cell subsets present in the SVF could partly interfere with the function of hASCs A recent study demonstrated that even modest adipose tissue expansion was characterized by a strong state of T lymphocyte activation. T lymphocyte activation was correlated with anti-inflammatory cytokine production likely associated with the presence of T regulatory cells [25]. The non-ASC cell components of the SVF could therefore play a role in modulating the function of hASCs.

The mechanisms by which hASCs may decrease fibrosis have been partly documented [26, 27]. Through the secretion of HGF or adrenomodullin, hASCs can reduce the expression of TGFβ1 and its target genes such as collagen type I, type III and α-Sma. Although only significant for TGF-β1,we observed the down-regulation of all these genes at day 8 after SVF injection, but not after hASC injection. However, hASCs induced a highly significant increase of TGFβ3 expression, resulting in a change in the TGFβ1/TGFβ3 ratio in favour of an anti-fibrotic effect. By contrast, hASCs tended to up-regulate MMP-1, -3 and significantly up-regulated MMP-2 and MMP-2/TIMP-2 ratio, while SVF cells did not. These results might suggest that cultured hASCs acted more likely on the remodelling of the fibrotic extracellular matrix while SVF cells regulated the TGFβ1 pathway. However, the uncoupling of these processes was unlikely. Another possible explanation might be a fast-acting effect of cultured hASCs upon injection, which was not detectable at the level of mRNAs 1 week after implantation. This was further supported by equivalent expression levels for the different genes, two weeks after implantation where the differences in skin thickness between the three groups of mice were the highest. Another possible mode of action could be through the modulation of miRNAs by hASCs. Such miRNAs, in particular miR-29a, has been reported to be decreased in bleomycin-induced fibrosis mouse model [28]. The possibility that hASCs could add by up-regulating miR-29a, which targets collagens and TIMPs, could not be excluded. Both SVF cells and hASCs have also been shown to exert an anti-apoptotic effect on the skin tissue [11, 29] The global effect of hASCs on healing was likely due to a balance between different modes of action, that still need to be further investigated.

Altogether, our results demonstrated the therapeutic efficacy of hASCs in a humanized model of HTS. Interestingly, both culture expanded hASCs and SVF cells exerted a similar anti-fibrotic effect on a pre-existing fibrous tissue, even though isolated hASCs seemed to reduce more efficiently the fibrotic scar. This study highlighted the therapeutic potential of hASCs for clinical applications of hypertrophic scarring.

## Supporting Information

S1 Fig Characterization of the SVF and hASCs by immunophenotyping. (A) Representative histograms of SVF immunophenotype as determined by multicolour staining and FACS analysis. Live cells in the freshly thawed SVF were gated according to the expression of CD45 antigen. Among the CD45^-^ cells, CD31^+^CD34^+^ endothelial cells and CD31^-^CD34^+^ hASCs were detected, while the CD45^+^ cells contained CD14^+^ macrophages and CD14^-^ lymphocytes. (B) Percentage of cells positive for the indicated markers in the CD45^-^CD31^-^CD34^+^ hASC population. (C) Average percentage of the different immune cell subsets in the SVF as shown in A). (D) Percentage of cells positive for the indicated CD markers in the ASC population obtained after expansion for 1 week in culture (end of P0).
(JPG)

S2 Fig Expression of fibrotic, remodelling and inflammatory markers after SVF or ASC injection in the hypertrophic scar humanized mouse model. Measure of mRNAs of the indicated genes normalized to human *YWHAZ* expression in skin samples at week 9. Results are expressed as the mean ± SEM (standard deviation of the mean), n = 5/group (one experiment representative of two). *p<0.05, **p<0.01.
(JPG)
