# Supplementary figures and images for: Comparison between Stromal Vascular Fraction and Adipose Mesenchymal Stem Cells in Remodeling Hypertrophic Scars
Source: PLoS One. 2016 May 26;11(5):e0156161. doi: 10.1371/journal.pone.0156161 (PMC4881943; doi:10.1371/journal.pone.0156161)

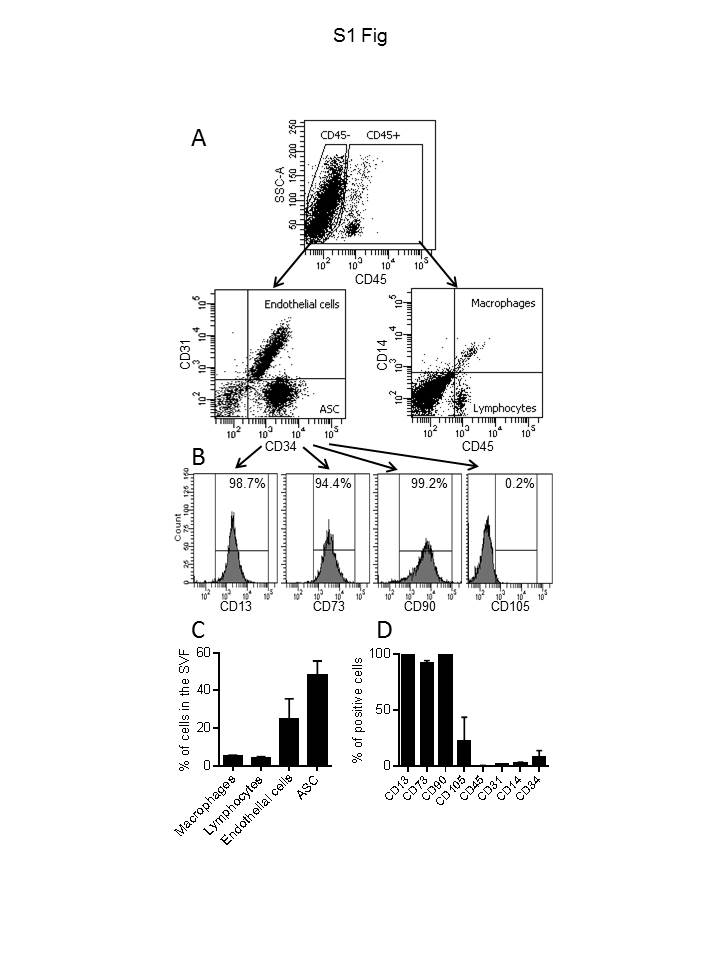

Supplement: S1 Fig — (A) Representative histograms of SVF immunophenotype as determined by multicolour staining and FACS analysis. Live cells in the freshly thawed SVF were gated according to the expression of CD45 antigen. Among the CD45- cells, CD31+CD34+ endothelial cells and CD31-CD34+ hASCs were detected, while the CD45+ cells contained CD14+ macrophages and CD14- lymphocytes. (B) Percentage of cells positive for the indicated markers in the CD45-CD31-CD34+ hASC population. (C) Average percentage of the different immune cell subsets in the SVF as shown in A). (D) Percentage of cells positive for the indicated CD markers in the ASC population obtained after expansion for 1 week in culture (end of P0). (JPG) [file pone.0156161.s001.JPG]

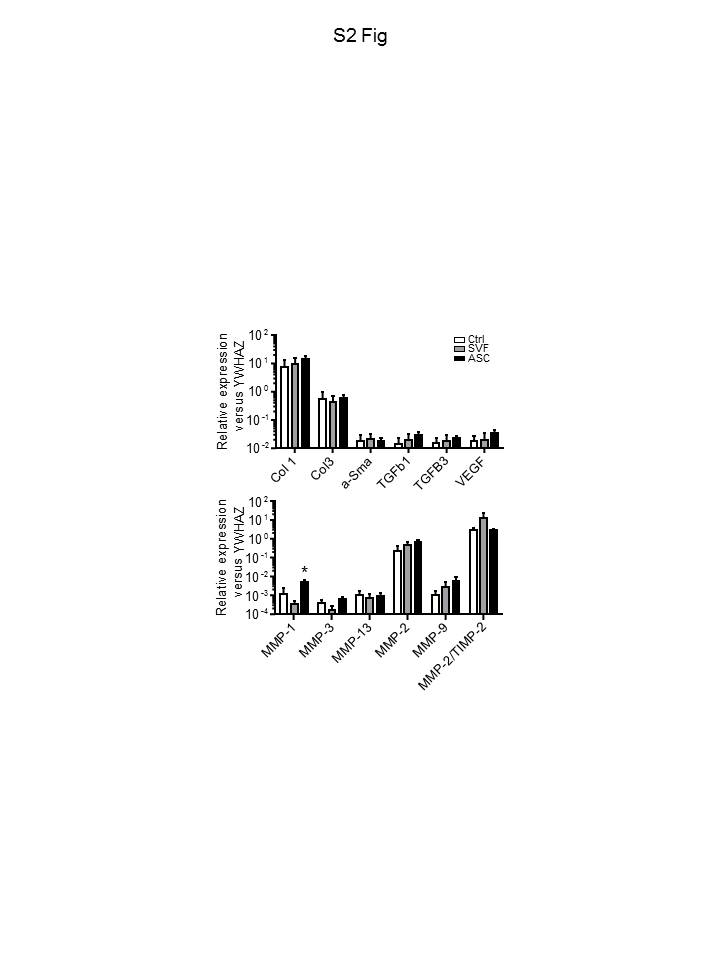

Supplement: S2 Fig — Measure of mRNAs of the indicated genes normalized to human YWHAZ expression in skin samples at week 9. Results are expressed as the mean ± SEM (standard deviation of the mean), n = 5/group (one experiment representative of two). *p<0.05, **p<0.01. (JPG) [file pone.0156161.s002.JPG]
